# Supplementary material for: Surface Microbiome Profiling of Dental Elevators Using Third‐Generation Sequencing: Implications for Infection Control in Dental Practice
Source: Microbiologyopen. 2025 Dec 8;14(6):e70178. doi: 10.1002/mbo3.70178 (PMC12685768; doi:10.1002/mbo3.70178)
Supplement: Supplementary file 1 — customer_backup. [file MBO3-14-e70178-s001.zip › customer_backup/customer_backup/taxa_summary/krona/samples/M09.Krona.html]

Javascript must be enabled to view this page.

members
magnitude
magnitudeUnassigned

M09.krona

60723

60421

3

3

3

2

2

2

1

1

1

1164

2

2

2

2

2

223

223

2

2

2

58

19

19

39

18

21

163

151

18

25

12

96

1

1

6

1

5

5

5

87

35

17

2

2

3

3

2

2

8

8

2

2

18

18

18

6

5

5

5

1

1

1

2

2

2

2

44

3

3

3

10

10

10

31

28

28

3

2

1

50

48

24

22

10

5

1

1

5

2

1

1

24

1

1

23

23

2

2

2

1

1

801

27

27

9

9

18

1

8

3

2

3

1

1

1

1

1

3

3

3

3

98

1

1

1

97

1

1

1

1

91

91

4

4

2

2

1

1

1

1

1

1

1

1

3

3

3

3

16

1

1

1

15

1

1

14

14

174

174

17

17

157

13

33

71

9

31

258

37

27

17

7

3

10

10

221

204

19

185

17

17

218

1

1

1

162

14

14

1

1

1

1

141

141

1

1

4

2

2

2

2

2

52

1

1

51

43

8

1

1

1

1

1

1

1

1

19

19

19

19

19

5

12

2

1

1

1

1

1

1

7

7

7

7

7

7

144

144

144

64

11

11

1

1

52

38

14

80

80

80

139

139

139

39

39

39

82

82

82

1

1

1

17

17

17

25

21

1

1

1

1

20

1

1

1

1

1

1

2

2

2

14

14

14

2

1

1

1

1

4

4

1

1

1

3

3

3

1

1

1

1

1

1

132

30

1

1

1

1

29

22

22

22

7

1

1

5

1

2

2

1

1

35

35

35

9

9

26

26

5

5

5

5

5

53

53

22

3

3

1

1

6

6

12

12

2

2

2

2

2

2

27

15

1

13

1

12

12

9

9

8

2

2

6

6

1

1

1

1

1

1

1

1

1

229

1

1

1

1

1

228

228

186

2

2

101

101

24

24

47

47

12

12

42

42

42

29

5

5

1

1

1

4

4

4

2

2

2

2

1

1

11

11

11

3

3

8

2

6

1

1

1

1

1

10

10

9

9

9

1

1

1

25

2

2

2

2

2

23

23

23

23

13

7

3

87

87

87

87

87

13

74

79

17

17

17

17

17

52

52

52

52

52

10

10

3

2

2

1

1

1

1

1

6

6

1

5

9

9

9

9

9

8

1

27609

5286

4

4

4

4

2

2

1

1

1

1

7

7

7

1

6

1

1

1

1

83

83

2

1

1

3

3

78

1

22

14

2

3

12

16

3

1

2

2

1

1

1

1

13

13

11

11

1

1

1

1

5128

7

7

7

3

2

2

1

1

14

13

3

10

1

1

106

103

103

3

3

4399

1

1

3

3

319

319

11

11

3052

3052

2

1

1

769

12

757

242

1

1

1

239

44

44

36

8

555

8

8

3

1

2

544

544

11

11

11

11

1

1

1

1

4

3

3

3

1

1

1

31

31

15

15

1

1

11

11

1

1

2

2

1

1

22323

1

1

1

1

14071

123

123

123

29

29

29

1

1

1

7848

7833

7833

11

2

2

5

2

4

1

1

2

2

1

1

1

1

51

11

11

40

40

5922

666

666

1

1

262

262

2

2

5

5

142

142

3989

3989

854

854

1

1

94

94

94

1

1

1

1881

9

4

4

5

5

2

2

2

12

12

12

1796

80

80

7

7

1709

1709

2

2

2

11

11

11

36

36

36

13

4

4

3

3

6

6

40

40

40

9

31

1

1

1

1

7

7

7

7

12

6

6

6

6

6

6

8

8

8

8

1831

2

1

1

1

1

1829

1

1

1824

1824

4

1

1

2

2

2

2

2

3

2

2

1

1

1

1

1

4380

4344

4344

65

3779

500

33

20

1

19

9

4

2

3

3

3

1

1

2

2

2

1

1

1

19

19

1

1

5

5

13

3

1

9

35

1

1

1

34

34

34

15

15

4

1

3

11

11

6

4

4

4

2

2

1

1

6

6

6

6

5

5

5

5

33

10

10

10

9

9

1

1

23

23

23

23

13

10

20437

13526

8

8

8

8

104

104

104

104

2

1

1

1

1

1

1

79

79

79

12

13

54

11163

11163

13

12

1

5

5

88

1

44

5

1

23

4

3

1

6

80

21

59

36

18

18

66

1

55

10

22

3

3

9

7

1408

13

11

37

53

958

333

3

164

103

61

10

10

947

9

51

848

39

246

246

58

58

21

21

1

1

31

31

42

42

108

17

90

1

20

20

89

89

2

2

25

25

105

5

100

794

511

3

26

2

220

9

8

2

13

6782

6696

77

9

315

95

13

13

40

40

42

42

204

3

3

39

39

162

162

16

16

16

45

45

20

18

1

1

25

25

74

6

6

6

12

12

12

56

56

56

1572

23

23

23

1015

95

95

1

1

81

1

20

60

3

3

15

15

342

156

1

114

33

38

14

14

138

138

23

3

20

283

67

1

215

20

1

19

39

39

39

470

139

117

22

47

47

213

212

1

14

14

41

41

1

1

15

15

25

9

9

16

16

164

57

57

57

27

27

27

71

71

71

9

9

9

5634

214

18

1

1

12

12

1

1

4

3

1

196

196

1

1

60

21

1

2

2

30

73

1

4

20

20

19

19

1

1

2662

208

35

35

164

5

1

157

1

1

1

1

1

7

7

2454

1409

1406

3

52

52

1

1

629

629

29

29

59

59

13

13

259

259

1

1

2

2

1

1

1

1

108

81

81

63

18

27

27

26

1

22

22

22

1

4

1

1

9

6

222

19

19

19

118

118

118

85

85

85

1

1

1

1

2361

8

8

7

1

10

10

10

340

334

1

39

13

148

22

32

36

29

2

12

6

6

5

5

1

4

1982

4

4

30

11

8

11

11

3

8

718

4

270

9

435

805

648

1

156

21

21

372

1

1

370

8

8

8

2

6

2

2

3

3

1

1

1

15

14

14

1

1

2

2

2

2

21

21

21

21

1277

1277

289

24

24

3

3

262

62

118

11

1

70

1

1

1

986

922

922

22

22

8

6

2

34

33

1

1

1

1

7

4

4

4

4

4

2

2

2

1

1

1

1

1

1

1

1

1

285

285

285

285

285

285

2

2

1

1

1

1

1

1

1

1

9950

9948

19

1

1

1

18

18

18

1726

59

1

1

4

4

36

1

21

14

6

6

1

1

1

1

4

4

1

1

2

2

1

1

2

2

5

5

5

7

7

7

1655

1530

1530

125

125

8192

1

1

1

60

60

60

6225

27

27

787

787

5411

5411

400

400

7

5

22

49

317

31

31

31

4

1

1

3

3

4

4

4

21

21

21

945

945

1

6

2

8

679

107

1

135

1

1

3

1

96

58

9

49

38

13

1

21

3

405

8

2

6

50

37

13

189

189

107

107

20

20

16

15

1

15

4

10

1

1

1

1

1

10

2

2

2

8

1

1

7

3

1

1

2

1

1

1

1

1

1

1

1

1

1

4

4

4

4

4

4

24

24

24

24

24

24

24

278

79

79

79

10

10

10

69

69

69

195

195

195

195

195

195

4

4

4

4

4

4
